# Efficacy of TAC-302 for patients with detrusor underactivity and overactive bladder: a randomized, double-blind, placebo-controlled phase 2 study

**DOI:** 10.1007/s00345-022-04163-4

**Published:** 2022-10-07

**Authors:** Masaki Yoshida, Momokazu Gotoh, Osamu Yokoyama, Hidehiro Kakizaki, Tomonori Yamanishi, Osamu Yamaguchi

**Affiliations:** 1 Department of Urology, Sakurajyuji Hospital, Kumamoto, Japan; 2 National Center for Geriatrics and Gerontology, Aichi, Japan; 3 Japan Community Health Care Organization Chukyo Hospital, Aichi, Japan; 4 Department of Urology, Harue Hospital, Fukui, Japan; 5 Department of Urology, University of Fukui, Fukui, Japan; 6 Department of Renal and Urologic Surgery, Asahikawa Medical University, Hokkaido, Japan; 7 Department of Urology, Continence Center, Dokkyo Medical University, Tochigi, Japan; 8 Fukushima Medical University, 1 Hikarigaoka, Fukushima-shi, Fukushima, 960-1295 Japan

**Keywords:** TAC-302, Detrusor underactivity, Underactive bladder, Overactive bladder, Bladder voiding efficiency

## Abstract

**Purpose:**

This multicenter, randomized, double-blind, placebo-controlled phase 2 study evaluated the efficacy and safety of TAC-302, a novel drug that restores neurite outgrowth, in patients with detrusor underactivity (DU) and overactive bladder (OAB).

**Methods:**

After 2–4 weeks of observation, patients were randomized 2:1 to receive oral TAC-302 200 mg or placebo twice daily for 12 weeks. The primary endpoint was detrusor contraction strength, estimated by bladder contractility index (BCI) for males and projected isovolumetric pressure 1 (PIP1) for females. Secondary endpoints included changes in bladder voiding efficiency (BVE) and safety.

**Results:**

Seventy-six patients were included (TAC-302, *n* = 52; placebo, *n* = 24). The mean (standard deviation [SD]) BCI for males was 64.6 (16.6) at baseline and 75.2 (21.1) at week 12 (*p* < 0.001) with TAC-302 (*n* = 27), and 61.3 (16.6) and 60.5 (16.7) (*p* = 0.82) with placebo (*n* = 11). The respective mean (SD) PIP1 for females was 18.8 (6.6) and 29.4 (9.4) (*p* < 0.001) with TAC-302 (*n* = 15), and 20.6 (7.5) and 25.5 (9.6) (*p* = 0.14) with placebo (*n* = 7). TAC-302 significantly increased BCI in males and BVE in both sexes. TAC-302 efficacy on OAB was not clearly shown. The incidences of adverse events (AEs), serious AEs, and AEs leading to dose interruption were similar between groups; no adverse drug reactions occurred.

**Conclusion:**

Considering the significant effects on BCI in males and BVE in both sexes, TAC-302 may benefit patients with DU.

**Registration:**

ClinicalTrials.gov Identifier NCT03175029 registered 6/5/2017.

**Supplementary Information:**

The online version contains supplementary material available at 10.1007/s00345-022-04163-4.

## Introduction

Detrusor hyperactivity with impaired contractility occurs in men and women, particularly among the elderly [1]. Urodynamically, this is described as storage-phase detrusor overactivity (DO) and voiding-phase detrusor underactivity (DU). Recent studies suggest the underlying mechanisms of bladder dysfunction may be chronic bladder ischemia and subsequent partial denervation [2]. In patients with DO, partial denervation occurs in the bladder, leading to overactive bladder (OAB) symptoms via denervation supersensitivity of the detrusor muscle. In addition, partial denervation reduces bladder contractility during the voiding phase, which is thought to lead to DU [3]. 

TAC-302, a novel and potent cyclohexenoic long-chain fatty alcohol derivative, promotes neurite outgrowth activity and was shown to improve storage and voiding dysfunctions by partially preventing bladder denervation in rats with bladder outlet obstruction [4]. Additionally, TAC-302 reduced the amplitude and frequency of non-voiding contractions. TAC-302 significantly decreased post-void residual (PVR) volume and increased bladder voiding efficiency (BVE) at a lower dose in rats [5]. These non-clinical studies suggest that TAC-302, which can restore neurite outgrowth, improves both storage and voiding function by suppressing partial denervation. Therefore TAC-302 may be a potential therapeutic agent for DU and DO. Given that DO is an etiologic factor for OAB, TAC-302 is expected to have a therapeutic effect on OAB symptoms. The present study was conducted to evaluate the efficacy and safety of TAC-302 in patients with both DU and OAB.

## Patients and methods

### Patients

Key inclusion criteria were ≥ 20 years of age, the presence of both voiding symptoms and OAB, a PVR ≤ 300 mL, and a diagnosis of DU. Voiding symptoms were confirmed if a patient had a voiding score of ≥ 2 points for at least one question on the International Prostatic Symptom Score (intermittency of voiding and weak stream and straining when voiding). OAB was confirmed using the Overactive Bladder Symptom Score (OABSS) (> 2 points for question 3 [urgency] and ≥ 3 points for the total score). DU was diagnosed using urodynamic testing (pressure flow study [PFS]) [6, 7]. Diagnostic criteria for males were bladder contractility index (BCI; maximum detrusor pressure at peak urine flow [*P*_det_*Q*_max_] + 5 peak urine flow rate [*Q*_max_]) < 100, bladder outlet obstruction index (*P*_det_*Q*_max_ − 2*Q*_max_) < 40, and BVE < 90%. Diagnostic criteria for females were *P*_det_*Q*_max_ < 20, *Q*_max_ < 15, and BVE < 90%.

Patients with clinically significant concurrent conditions, cystocele ≥ stage 3 (females), prostate gland volume ≥ 30 mL (males), overflow urinary incontinence, urethral catheter placement, or clean intermittent self-catheterization were excluded.

### Study design and treatments

This was a multicenter, randomized, double-blind, placebo-controlled phase 2 study. All patients received oral placebo twice daily during the single-blind observation period (2–4 weeks). Patients were then randomized 2:1 to receive oral TAC-302 200 mg or placebo twice daily for 12 weeks (double-blind treatment period; evaluations at 2, 4, 8, and 12 weeks), followed by a 1-week follow-up period (Online Resource 1). Dynamic assignment randomization was conducted by central registration using an Interactive Web Response System; randomization factors were sex (male/female) and age at informed consent (< 65 years/ ≥ 65 years). Key codes retained double-blinding and were stored and managed by the investigational drug assignment manager. The principal investigator or investigators at each study site enrolled patients via the Interactive Web Response System; treatment compliance was monitored at study visits using drug diaries, empty study drug containers, and patient interviews. The study protocol was approved by an Institutional Review Board at each participating institution. The study was conducted in accordance with the ethical principles outlined in the Declaration of Helsinki, the Pharmaceutical and Medical Device Act, and the ministerial ordinance on Good Clinical Practice. All patients provided written informed consent. This study was registered at ClinicalTrials.gov (NCT03175029).

### Efficacy endpoints

The primary efficacy endpoint was the mean BCI for males and projected isovolumetric pressure 1 (PIP1; *P*_det_*Q*_max_ + *Q*_max_) for females [8] at baseline and week 12. Secondary endpoints included changes in BVE, mean number of micturitions per 24 h, mean number of urgency episodes per 24 h, and OABSS total score from baseline to week 12.

### Safety assessments

Safety assessments included adverse events (AEs), vital signs, 12-lead electrocardiogram, laboratory tests, and PVR urine volume. AEs were coded using the Medical Dictionary for Regulatory Activities (MedDRA), version 22.1.

### Statistical methods

The primary analysis of this study is to calculate summary statistics and 95% confidence intervals for each treatment group based on the time point of evaluation. PFS is an invasive test and evaluation is complex, limiting the number of study sites able to participate; therefore, the sample size of 75 patients was determined based on feasibility. The full analysis set (FAS) included all patients who took the study drug at least once and had ≥ 1 efficacy measurement before and during the treatment period. The per protocol set (PPS) included all patients in the FAS with no inclusion/exclusion criteria violations, who were compliant for ≥ 80% of the treatment period, were not administered any prohibited concomitant medication or therapy during the study period, and had a week 12 evaluation of the primary endpoint. The FAS and PPS were used for efficacy evaluations. The PPS was the primary analysis population for the primary evaluation measure. Safety was assessed in all treated patients. 

Categorical variables were summarized using descriptive statistics. Efficacy variables were analyzed using paired Student’s *t*-tests, summary statistics, and 95% confidence intervals (CIs). Treatment groups were compared using Student’s *t*-test and analysis of covariance (ANCOVA) (change in BCI [males]/PIP1 [females] from baseline to 12 weeks); allocation group, baseline value, and age (≥ 60 or < 60 years) were set as covariates. Statistical analyses were performed using SAS statistical software (version 9.4; SAS Institute Inc., Cary, NC, USA).

## Results

### Patients

This study was conducted at 21 medical institutions in Japan and the study period was from September 9, 2017 to November 14, 2019 were randomized to receive TAC-302 (*n* = 52) or placebo (*n* = 24) (FAS) (Online Resource 2). A respective 3 and 1 patient(s) discontinued and 49 and 23 patients completed the study. The PPS population included 42 patients in the TAC-302 group (male, *n* = 27; female, *n* = 15) and 18 in the placebo group (male, *n* = 11; female, *n* = *7*). The mean patient age was 70.8 years, mean baseline BCI values in the TAC-302 and placebo groups were 64.6 and 61.3, and mean baseline PIP1 values were 18.8 and 20.6 (Table 1), respectively.

**Table 1:** Baseline demographics and clinical characteristics

	Total, *N* = 60	TAC-302, *n* = 42	Placebo, *n* = 18
Gender			
Male, *n* (%)	38 (63.3)	27 (64.3)	11 (61.1)
Female, *n* (%)	22 (36.7)	15 (35.7)	7 (38.9)
Age (years)			
Mean (SD)	70.8 (10.2)	71.0 (10.7)	70.4 (9.1)
Median	74.0	74.0	73.5
Duration of lower urinary tract symptoms (months)			
Mean (SD)	86.4 (88.4)	92.2 (95.1)	72.9 (71.1)
Median	68.0	79.0	45.5
BVE (%)			
Mean (SD)	59.8 (25.9)	58.4 (26.7)	63.1 (24.1)
Median	69.2	65.2	73.5
BCI (males only)			
Mean (SD)	63.7 (16.4)	64.6 (16.6)	61.3 (16.6)
Median	68.0	68.0	60.0
PIP1 (females only)			
Mean (SD)	19.3 (6.8)	18.8 (6.6)	20.6 (7.5)
Median	17.9	17.0	21.0

*BCI* bladder contractility index, *BVE* bladder voiding efficiency, *PIP1* projected isovolumetric pressure 1, *SD* standard deviation

### Efficacy

**Fig. 1:**
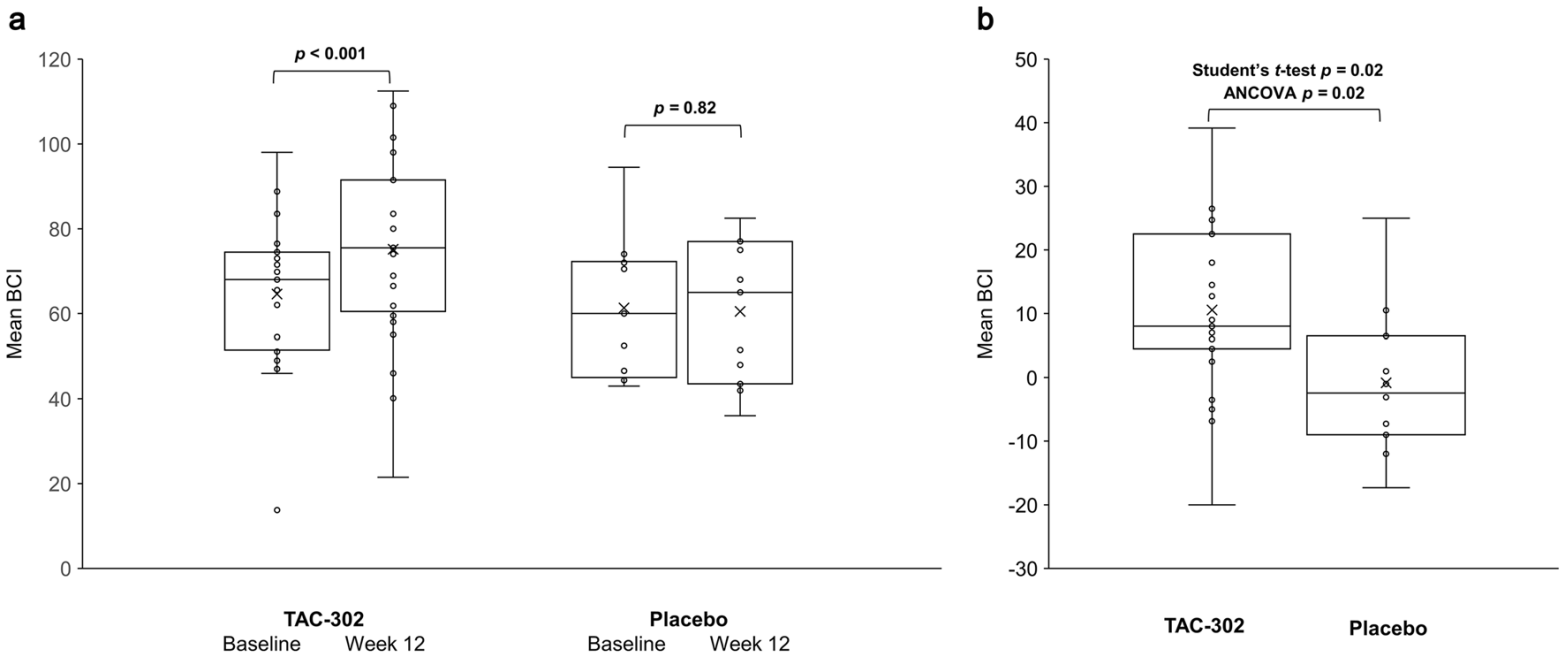
**a** Shows the results for increased BCI in male patients before and after treatment in each group. *p* values for baseline vs week 12 were calculated using the Paired *t* test. In contrast, Fig. 1b compares the difference between the TAC-302 group and the placebo group in the amount of change in BCI at 12 weeks. *p* values for BCI change from baseline for TAC-302 vs placebo were calculated using both the Student’s *t* test and ANCOVA. Figures 2a, b, as in **a** and **b**, show the evaluation of PIP1 in female patients. The horizontal line in the middle of each box indicates the median; the × indicates the mean; the ○ indicates individual data; the top and bottom borders of the box mark the 75th and 25th percentiles, respectively. The whiskers above and below the box extend to the data point furthest from the box that is still within 1.5 × (75th percentile to 25th percentile) the box. *ANCOVA* analysis of covariance, *BCI* bladder contractility index

**Fig. 2:**
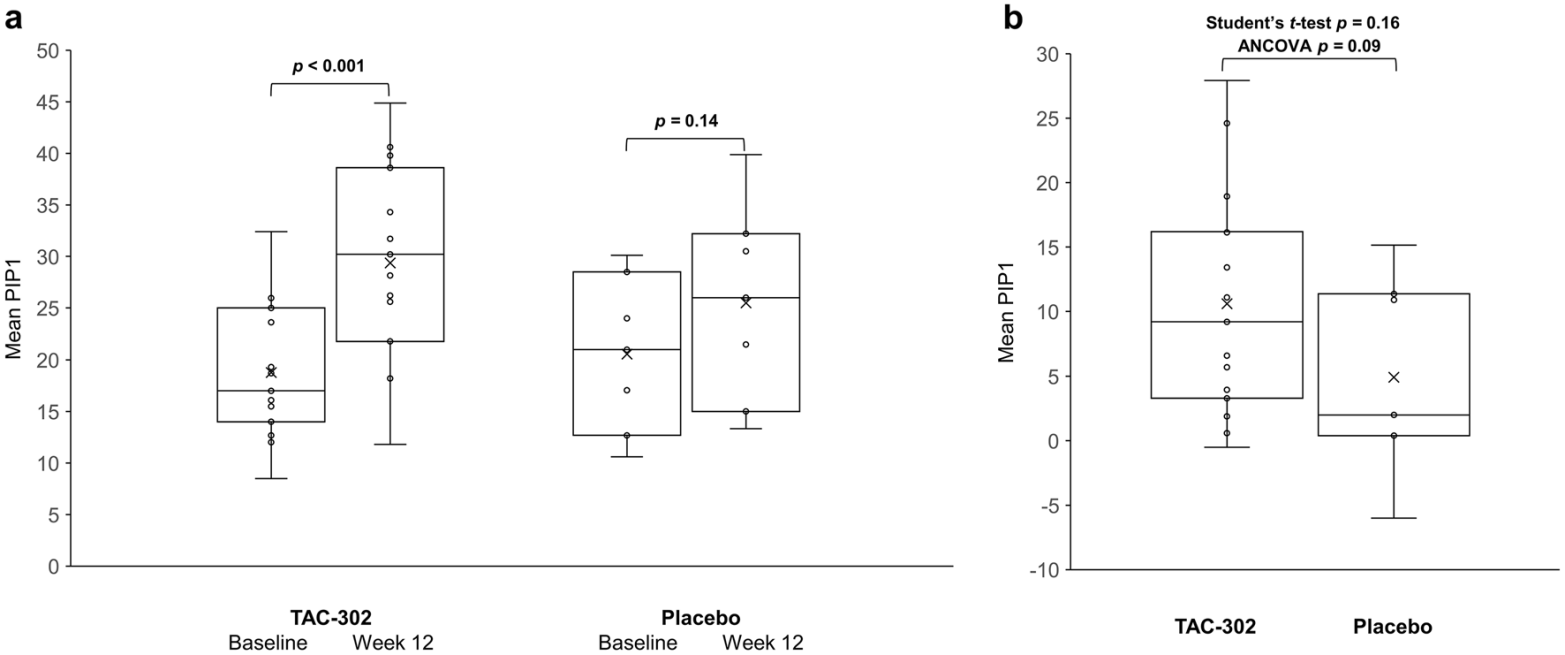
**a** PIP1 at baseline and week 12. **b** Change in PIP1 (females only) from baseline. The horizontal line in the middle of each box indicates the median; the × indicates the mean; the ○ indicates individual data; the top and bottom borders of the box mark the 75th and 25th percentiles, respectively. The whiskers above and below the box extend to the data point furthest from the box that is still within 1.5 × (75th percentile to 25th percentile) the box. *p* values for baseline *vs* week 12 were calculated using the Paired *t* test, and *p* values for PIP1 change from baseline for TAC-302 *vs* placebo were calculated using both the Student’s *t*-test and ANCOVA. *ANCOVA* analysis of covariance, *PIP1* projected isovolumetric pressure 1

The mean BCI (primary endpoint, males) increased from 64.6 (standard deviation [SD] 16.6; 95% CI 58.1, 71.2) at baseline to 75.2 (SD 21.1; 95% CI 66.8, 83.5) at week 12 (mean change 10.6; *p* < 0.001) in the TAC-302 group (*n* = 27) and did not significantly change in the placebo group (*n* = 11) (baseline 61.3 [SD 16.6; 95% CI 50.2, 72.5]; week 12 60.5 [SD 16.7; 95% CI 49.3, 71.7]; mean change − 0.83; *p* = 0.82) (Fig. 1a). BCI was significantly increased in the TAC-302 group compared with placebo (mean difference 11.4; Student’s *t* test *p* = 0.02; ANCOVA *p* = 0.02) (Fig. 1b). The mean PIP1 (primary endpoint, females) increased from 18.8 (SD 6.6; 95% CI 15.1, 22.4) at baseline to 29.4 (SD 9.4; 95% CI 24.2, 34.6) at week 12 (*p* < 0.001) in the TAC-302 group (*n* = 15) and did not change significantly in the placebo group (*n* = 7) (baseline 20.6 [SD 7.5; 95% CI 13.6, 27.5]; week 12 25.5 [SD 9.6; 95% CI 16.6, 34.3]; *p* = 0.14) (Fig. 2a). The increase in PIP1 in the TAC-302 group was not significant compared to the placebo group (mean difference 5.68; Student’s *t* test *p* = 0.16; ANCOVA *p* = 0.09) (Fig. 2b).

**Fig. 3:**
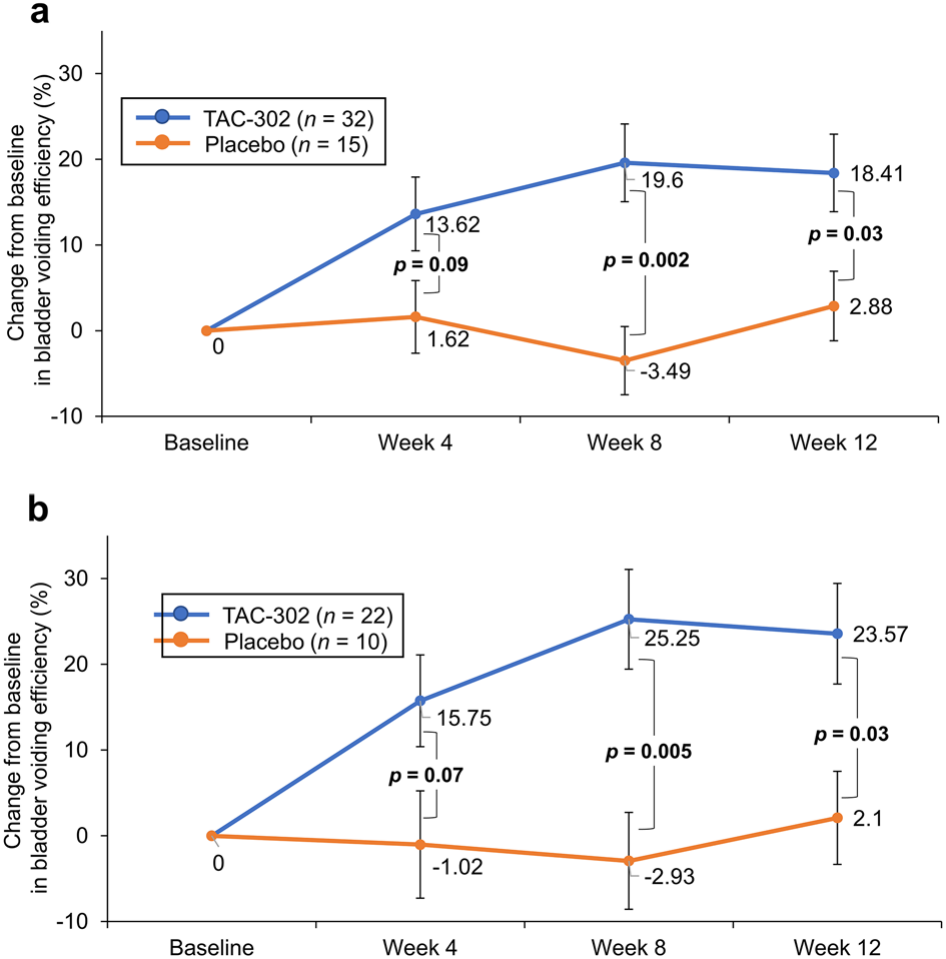
Change from baseline in BVE according to baseline post void residual volume: **a** ≥ 50 mL, **b** ≥ 100 mL (FAS). *P*-values were calculated using the Student’s *t* test (TAC-302 *vs* placebo). *BVE* bladder voiding efficiency, *FAS* full analysis set

Efficacy was observed for TAC-302 relative to the placebo group for BVE, which tended to increase at week 12 (*p* = 0.006) with TAC-302 but had no obvious increase with placebo (*p* = 0.57) (FAS). Subgroup analysis demonstrated a significant difference between groups at week 12 (*p* = 0.03) (Fig. 3a, b). The mean number of micturitions per 24 h, number of urgency episodes per 24 h, and total OABSS were not different between the treatment groups (Online Resource 3).

### Safety

The incidences of any AE were similar between the groups (TAC-302, 46.2%; placebo, 37.5%) (Table 2). AEs occurring in ≥ 2 patients during the treatment period were diarrhea, pyrexia, nasopharyngitis, pyuria, enteritis infectious, back pain, and headache (3.8% [2/52] each) in the TAC-302 group, and constipation (8.3% [2/24]) in the placebo group. Incidences of serious AEs and AEs leading to dose interruption were similar between groups (TAC-302, 3.8% [2/52] each; placebo, 4.2% [1/24] each). No adverse drug reactions occurred in either group; there was no obvious difference in the incidence of AEs by age.

**Table 2:** AEs occurring in ≥ 2% of patients during the treatment period (all treated patients)

	TAC-302, *n* = 52	Placebo, *n* = 24
Any AE	24 (46.2)	9 (37.5)
Deafness neurosensory	0	1 (4.2)
Constipation	1 (1.9)	2 (8.3)
Dental caries	0	1 (4.2)
Diarrhea	2 (3.8)	0
Diverticulum intestinal hemorrhagic	0	1 (4.2)
Glossitis	0	1 (4.2)
Hematochezia	0	1 (4.2)
Nausea	0	1 (4.2)
Vomiting	0	1 (4.2)
Pyrexia	2 (3.8)	0
Bacteriuria	1 (1.9)	0
Bronchitis	1 (1.9)	1 (4.2)
Cystitis	1 (1.9)	1 (4.2)
Gastroenteritis	1 (1.9)	0
Herpes virus infection	0	1 (4.2)
Influenza	1 (1.9)	0
Nasopharyngitis	2 (3.8)	1 (4.2)
Periodontitis	0	1 (4.2)
Pharyngitis	1 (1.9)	1 (4.2)
Pyuria	2 (3.8)	0
Urinary tract infection	1 (1.9)	0
Vulvitis	1 (1.9)	0
Enteritis infectious	2 (3.8)	0
Enterocolitis viral	1 (1.9)	0
Compression fracture	1 (1.9)	0
Fracture	1 (1.9)	0
Subdural hematoma	1 (1.9)	0
Contusion	1 (1.9)	0
Post procedural hematuria	1 (1.9)	0
Meniscus injury	1 (1.9)	0
Tooth dislocation	1 (1.9)	0
Blood creatinine phosphokinase increased	1 (1.9)	0
Back pain	2 (3.8)	0
Pain in extremity	0	1 (4.2)
Headache	2 (3.8)	0
Dysuria	1 (1.9)	0
Renal colic	1 (1.9)	0
Urethral pain	1 (1.9)	0
Prostatitis	1 (1.9)	0
Cough	0	1 (4.2)
Dermatitis contact	0	1 (4.2)
Miliaria	1 (1.9)	0
Rash	0	1 (4.2)
Orthostatic hypotension	0	1 (4.2)
Any adverse drug reaction	0	0
Any serious AE	2 (3.8)	1 (4.2)
Prostatitis	1 (1.9)	0
Subdural hematoma	1 (1.9)	0
Diverticulum intestinal hemorrhagic	0	1 (4.2)
AE leading to discontinuation	0	0
AE leading to death	0	0
AE leading to dose interruption	2 (3.8)	1 (4.2)
Enteritis infectious	1 (1.9)	0
Enterocolitis viral	1 (1.9)	0
Diverticulum intestinal hemorrhagic	0	1 (4.2)

Data are *n* (%)

*AE* adverse event

## Discussion

This study does not design the sample size based on hypothesis test setting. PFS is an invasive test, which decreases the number of study sites and participant patients. Therefore, the sample size of 75 patients was determined based on feasibility. At the end of the study, the 76th patient's consent was fortuitously obtained and enrolled. This may be one of limitations of the current study. This phase 2 study demonstrated a significant increase in BCI (males) with TAC-302 treatment versus placebo, but not in PIP1 (females). A likely explanation is that fewer female (PPS: TAC-302, *n* = 15; placebo, *n* = 7) than male (PPS: TAC-302, *n* = 27; placebo, *n* = 11) patients were included. ANCOVA analysis revealed a tendency towards a difference between TAC-302 and placebo for PIP1. The effect of TAC-302 on the secondary endpoints, especially the change in BVE, was more evident in the FAS than the PPS population. Patients in the FAS were excluded from the PPS because of missing data related to indicators of detrusor muscle strength (PFS parameters) either before or after treatment administration. Therefore, data from the FAS could not be used to determine the primary endpoint. Secondary endpoint measurements were obtained from patients in the FAS; given the larger patient number and increased statistical power versus the PPS, the FAS was most appropriate for secondary endpoint evaluations.

BVE is a reliable, highly reproducible parameter for evaluating emptying function [9]. The significant changes in BVE from baseline to week 12 suggest that TAC-302 may be efficacious, especially in patients with a baseline PVR of ≥ 50 mL or ≥ 100 mL. In this study, PVR was not evaluated because it has been shown to depend on bladder volume prior to voiding [9].

Our non-clinical study showed that 4-week oral treatment of TAC-302 significantly improved detrusor underactivity in rats with bladder outlet obstruction (BOO) [10]. Since BOO induces denervation of the urinary bladder, it is assumed that TAC-302 improves detrusor underactivity by partially preventing bladder denervation. This may have led to beneficial clinical efficacy within 4 weeks.

Efficacy of TAC-302 for OAB symptoms was expected; however, this was not observed in the present study. The TAC-302 dose used may have been insufficient for evaluation of storage function parameters. Using a rat model of impaired urination due to bladder outlet obstruction, TAC-302 significantly decreased PVR and non-voiding contractions, and increased BVE. However, the TAC-302 dose that significantly decreased non-voiding contractions (e.g., effect on DO) was 10 times higher [4]. We think that the lack of a significant effect of TAC-302 on OAB symptoms may be explained by the fact that the lower dosage of TAC-302 was used in the present clinical study.

No adverse drug reactions were reported, indicating that TAC-302 can be safely administered for a period of 12 weeks. Given that the study evaluations included invasive PFS testing, which involved catheterization, it was possible for patients to experience related pain, hematuria, or urinary tract infections. Despite this, such AEs were not problematic.

While the present study suggests that TAC-302 may have a therapeutic effect on DU, the patient number was limited. The sample size was determined based on feasibility of evaluations rather than statistical analysis. Additionally, this study was conducted only in Japanese patients, limiting the generalizability of the findings. Further studies are required to elucidate the therapeutic effect of TAC-302.

## Supplementary Information

Below is the link to the electronic supplementary material.

Supplementary file1 (DOCX 207 KB)

## Declarations

### Data sharing statement

The policy on data sharing of the study sponsor, Taiho Pharmaceutical Co., Ltd. may be found at https://www.taiho.co.jp/en/science/policy/clinical_trial_information_disclosure_policy/index.html.
