# Supplementary material for: Efficacy of TAC-302 for patients with detrusor underactivity and overactive bladder: a randomized, double-blind, placebo-controlled phase 2 study
Source: World J Urol. 2022 Oct 7;40(11):2799–805. doi: 10.1007/s00345-022-04163-4 (PMC9617838; doi:10.1007/s00345-022-04163-4)
Supplement: Supplementary file 1 — Supplementary file1 (DOCX 207 KB) [file 345_2022_4163_MOESM1_ESM.docx]

**Efficacy of TAC-302 for patients with detrusor underactivity and overactive bladder: A randomized, double-blind, placebo-controlled phase 2 study**

**Journal name**: World Journal of Urology

Masaki Yoshida^1^, Momokazu Gotoh^2^, Osamu Yokoyama^3^, Hidehiro Kakizaki^4^, Tomonori Yamanishi^5^, Osamu Yamaguchi^6^

^1^Department of Urology, Sakurajyuji Hospital, Kumamoto, Japan and National Center for Geriatrics and Gerontology, Aichi, Japan

^2^Chukyo Hospital, Aichi, Japan

^3^Department of Urology, University of Fukui Faculty of Medical Sciences, Fukui, Japan

^4^Department of Urology, Asahikawa Medical University, Hokkaido, Japan

^5^Department of Urology, Continence Center, Dokkyo Medical University, Tochigi, Japan

^6^Fukushima Medical University, Fukushima, Japan

**Corresponding author:**

Osamu Yamaguchi

Fukushima Medical University

1 Hikarigaoka, Fukushima-shi, Fukushima, 960-1295, Japan

Email: oyamagu52@gmail.com

**Online Resource 1** Study design


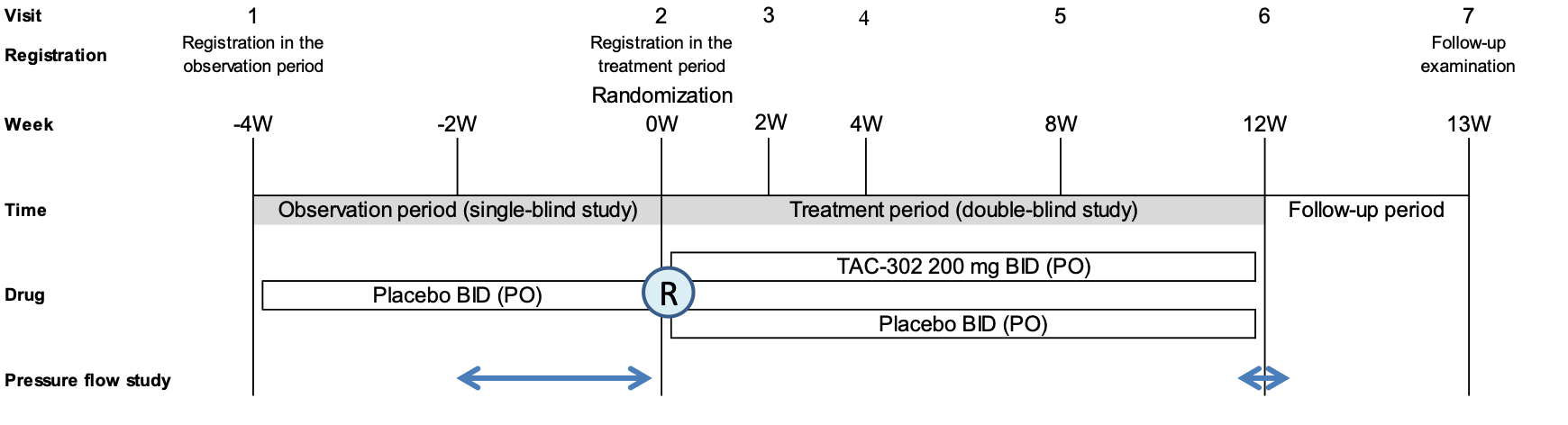


BID, twice daily; PO, per oral; R, randomization; W, week

**Online Resource 2** Patient disposition

**
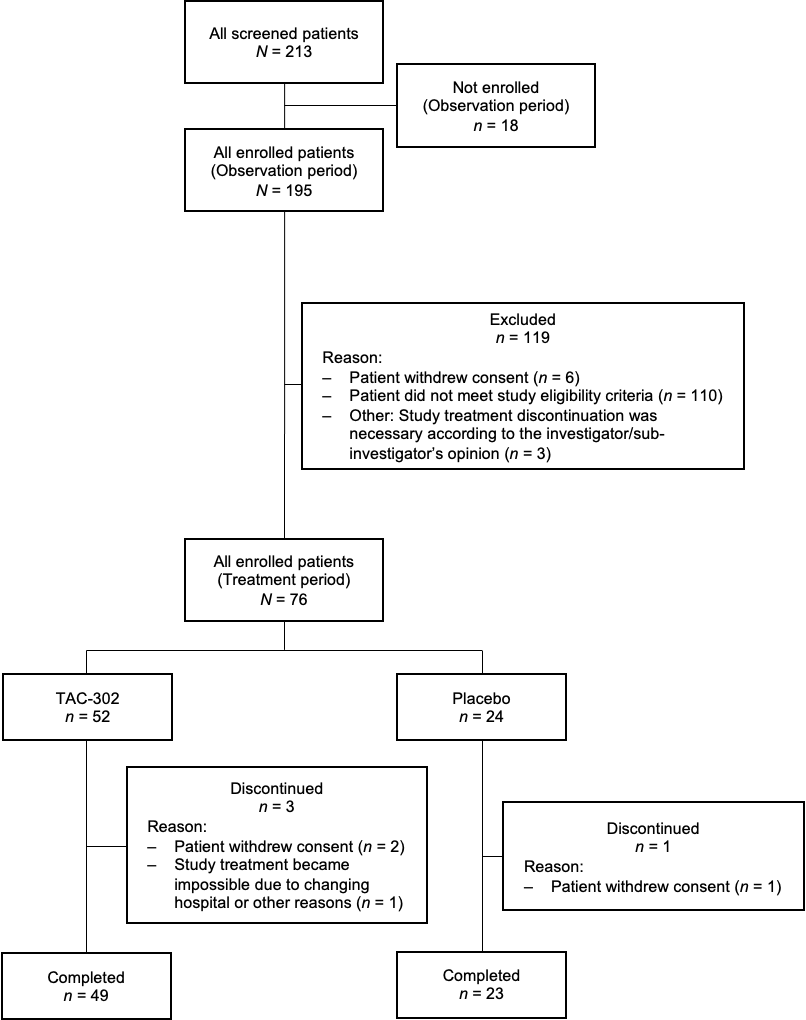
**

**Online Resource 3** Storage symptoms

|  | TAC-302 | Placebo |
| --- | --- | --- |
| Micturitions per 24 h (average) |  |  |
| Baseline | *n =* 52 | *n =* 24 |
| Mean (SD) | 11.8 (3.1) | 11.8 (3.1) |
| Week 12 | *n =* 49 | *n =* 23 |
| Mean (SD) | 10.8 (4.0) | 10.2 (2.3) |
| Urinary urgency episodes per 24 h (average) |  |  |
| Baseline | *n =* 52 | *n =* 24 |
| Mean (SD) | 5.5 (4.6) | 5.7 (3.9) |
| Week 12 | *n =* 49 | *n =* 23 |
| Mean (SD) | 4.5 (6.1) | 2.5 (3.6) |
| OABSS total score |  |  |
| Baseline | *n =* 52 | *n =* 24 |
| Mean (SD) | 8.7 (2.9) | 8.9 (2.4) |
| Week 12 | *n =* 49 | *n =* 23 |
| Mean (SD) | 6.5 (3.2) | 7.3 (3.0) |

OABSS, overactive bladder symptom score; SD, standard deviation
